# A Comparison of the Safety and Efficacy of Remimazolam and Dexmedetomidine for Sedation in Surgical Patients Under Regional Anesthesia: A Meta-Analysis of Randomized Controlled Trials

**DOI:** 10.3390/medicina61040726

**Published:** 2025-04-14

**Authors:** Hyo-Seok Na, Sang-Hi Park, Bon-Wook Koo, Seunguk Bang, Hyun-Jung Shin

**Affiliations:** 1 Department of Anesthesiology and Pain Medicine, Seoul National University Bundang Hospital, Seongnam 13620, Republic of Korea; 2 Department of Anesthesiology and Pain Medicine, Seoul National University College of Medicine, Seoul 03080, Republic of Korea; 3 Department of Anesthesiology and Pain Medicine, Chungbuk National University Hospital, Cheongju-si 28644, Republic of Korea; 4 Department of Anesthesiology and Pain Medicine, Chungbuk National University College of Medicine, Cheongju-si 28644, Republic of Korea; 5 Department of Anesthesiology and Pain Medicine, Daejeon St. Mary’s Hospital, The Catholic University of Korea College of Medicine, Daejeon 34943, Republic of Korea

**Keywords:** remimazolam, dexmedetomidine, respiratory depression, hypotension, bradycardia, meta-analysis

## Abstract

*Background and Objectives*: This meta-analysis compares the safety and efficacy of remimazolam and dexmedetomidine for sedation during regional anesthesia, focusing on respiratory and hemodynamic outcomes. *Materials and Methods*: A systematic search of CENTRAL, Embase, PubMed, Scopus, and Web of Science up to November 2024 identified randomized controlled trials (RCTs) comparing remimazolam with dexmedetomidine. Outcomes included respiratory depression (primary outcome), bradycardia, hypotension, hypertension, respiratory and heart rates, mean arterial pressure, sedation onset time, emergence time, and postoperative nausea and vomiting (PONV). Effect sizes were calculated as relative risks (RRs) or mean differences (MDs) using random-effects models. *Results*: Five RCTs involving 439 participants were included. Remimazolam did not significantly increase respiratory depression risk compared to dexmedetomidine (RR: 1.36, 95% CI [0.39, 4.71], *p* = 0.6305, I^2^ = 44%). Bradycardia incidence was lower with remimazolam (RR: 0.15, 95% CI [0.06, 0.39], *p* = 0.0001, I^2^ = 0%). Remimazolam showed faster sedation onset (MD: −6.04 min, 95% CI [−6.99, −5.09], *p* = 0.0000, I^2^ = 68%). Both drugs demonstrated similar occurrences of hypotension and hypertension, respiratory rates, mean arterial pressures, emergence times, and incidences of PONV. *Conclusions*: Remimazolam offers comparable safety and efficacy to dexmedetomidine, with advantages such as lower bradycardia risk and faster sedation onset. These findings support remimazolam as a viable sedative option during regional anesthesia, although further large-scale studies are warranted to confirm these results and optimize sedation practices.

## 1. Introduction

Sedation is a critical component of regional anesthesia, ensuring patient comfort and safety during surgical procedures [1]. An ideal sedative agent is expected to provide rapid and reliable onset of sedation, maintain hemodynamic stability, and minimize respiratory depression and other adverse events [2]. Additionally, it is desirable for the agent to facilitate prompt recovery and reduce the incidence of postoperative complications such as nausea and vomiting [3]. While achieving all these required characteristics remains challenging, emerging agents like remimazolam and established options like dexmedetomidine have shown promise in meeting these criteria in specific clinical contexts.

Remimazolam, a novel benzodiazepine, has recently emerged as a promising agent for procedural sedation due to its rapid onset, short duration of action, and favorable safety profile [4]. Dexmedetomidine, an alpha-2 adrenergic receptor agonist, is widely used for its sedative, anxiolytic, and mild analgesic properties but is also known for hemodynamic effects such as bradycardia, hypotension, and hypertension [5]. While both agents are valuable sedatives, their distinct pharmacological profiles necessitate a direct comparison to identify the most appropriate choice for specific surgical settings, particularly for patients undergoing regional anesthesia, where ensuring patient safety and optimizing perioperative care are of critical importance.

This meta-analysis is designed to synthesize the existing evidence from randomized controlled trials (RCTs) to compare remimazolam and dexmedetomidine in terms of respiratory and hemodynamic outcomes, sedation efficacy, and adverse events. By systematically evaluating the available data, this study aims to provide clinicians with a comprehensive understanding of the advantages and limitations of these two sedative agents, facilitating informed decision-making in the perioperative setting.

## 2. Materials and Methods

This meta-analysis was planned and documented following the guidelines outlined in the 2020 PRISMA (Preferred Reporting Items for Systematic Reviews and Meta-Analyses) statement. Before commencing the research, the study protocol was registered with PROSPERO (International Prospective Register of Systematic Reviews) and assigned the registration number “CRD42024622691”.

### 2.1. Eligible Criteria

The selection of studies was guided by predefined PICOS criteria (population, intervention, comparison, outcomes, and study design): P, adult participants receiving regional anesthesia with sedation; I, administration of remimazolam; C, comparison with dexmedetomidine; O, respiratory and hemodynamic outcomes; and S, RCTs reporting at least one relevant outcome. Studies that did not meet these criteria, such as protocols, conference abstracts, editorials, case reports, review articles, observational studies, or retrospective analyses, were excluded from the analysis.

### 2.2. Search Strategy

Eligible RCTs were identified through a comprehensive search of electronic databases, including CENTRAL, Embase, PubMed, Scopus, and Web of Science, covering the period from inception to 27 November 2024. The search imposed no restrictions on language, journal, region, or publication year. Keywords used in the search included “Remimazolam”, “Byfavo”, “CNS-7056”, “Dexmedetomidine”, “Precedex”, “Dexdor”, “Dexdomitor”, “Dexmed”, “Dexem”, “Dexit”, and “sedation”, which were combined with Boolean operators to maximize the retrieval of relevant studies across databases. Details of the search strategy are provided in Supplementary Table S1.

### 2.3. Study Selection and Data Extraction

The studies retrieved from all databases were combined, and the selection of eligible RCTs was independently performed by two investigators (HSN and HJS). The process involved removing duplicate entries, screening titles and abstracts, and subsequently reviewing the full texts of potentially relevant studies. The final set of RCTs included in the data synthesis was determined after a thorough evaluation of the full-text articles. Discrepancies between the two investigators were resolved through consultation with a third investigator (BWK).

Essential variables were extracted from the included RCTs, systematically organized, and documented in a data sheet. The collected information comprised the year of publication, authors, number of participants, age, surgical type, doses of remimazolam and dexmedetomidine used for sedation induction and maintenance, and outcomes such as respiratory depression, bradycardia, hypotension, hypertension, respiratory rates, heart rates, mean arterial pressure, time to achieve the target sedation depth, emergence time, and postoperative nausea and vomiting (PONV). Median values with interquartile ranges were transformed into means and standard deviations using Wan’s formula [6]. Data represented in graphical formats were extracted using WebPlotDigitizer (https://apps.automeris.io/wpd/, assessed on 4 December 2024).

### 2.4. Outcome Measures

The primary outcome was the incidence of respiratory depression during sedation. Secondary outcomes included the occurrence of bradycardia, hypotension, and hypertension; respiratory rates; heart rates; mean arterial pressure during sedation; and the incidence of postoperative nausea and vomiting (PONV).

### 2.5. Assessment of Risk of Bias and Quality of Evidence

The risk of bias for each outcome variable was evaluated independently by two investigators (HSN and HJS) using the updated Cochrane risk of bias tool for randomized trials (RoB 2) [7]. The evaluation focused on five key domains: (1) bias from the randomization process, (2) bias due to deviations from intended interventions, (3) bias resulting from incomplete outcome data, (4) bias in outcome measurement, and (5) bias related to the selection of reported results. Each domain was classified as having “low risk”, “some concerns”, or “high risk” of bias.

The certainty of evidence for each outcome was determined using the Grading of Recommendations, Assessment, Development, and Evaluation (GRADE) framework [8]. This method considers five critical factors: (1) the risk of bias within the included studies, (2) inconsistency in results across studies, (3) the directness of the evidence in addressing the research question, (4) the precision of effect estimates, and (5) the potential impact of publication bias on the conclusions.

### 2.6. Statistical Analysis

Effect sizes for each study were calculated using Stata^®^ SE version 17.0 (Stata Corp., College Station, TX, USA) to evaluate the impact of the interventions. For binary outcomes, the effect size was expressed as the relative risk (RR) along with a 95% confidence interval (CI), while for continuous outcomes, the mean difference (MD) was used, also with a 95% CI. A random-effects model was applied across all statistical analyses to account for variability between studies, yielding a more reliable estimate of the effect size. Statistical significance was set at a *p*-value of less than 0.05.

To assess heterogeneity among the pooled effect sizes, Cochran’s Q test and the I^2^ statistic were used, with I^2^ reflecting the proportion of variability attributable to genuine heterogeneity rather than random fluctuation. Heterogeneity was classified into the following levels: none (I^2^ < 25%), low (I^2^ = 26–49%), moderate (I^2^ = 50–74%), and high (I^2^ > 75%). A leave-one-out sensitivity analysis was conducted to examine the potential small-study effect by evaluating whether the exclusion of any individual study significantly altered the pooled effect size. In accordance with Cochrane guidelines [9], publication bias was not evaluated visually with a funnel plot or statistically with Egger’s test if fewer than 10 studies were available for each outcome.

## 3. Results

### 3.1. Study Selection

**Figure 1:**
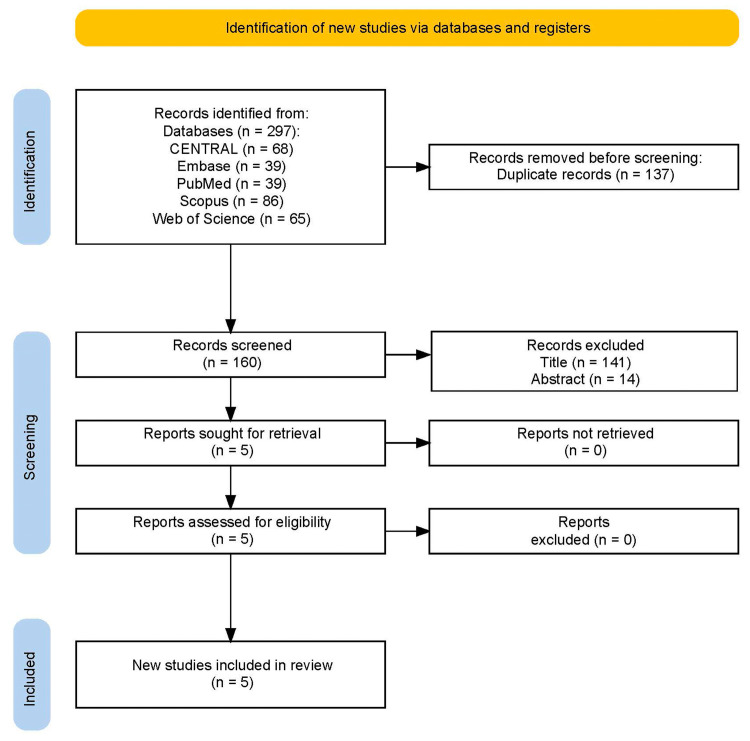
Flow diagram of study selection.

Through electronic database searches, 297 records were retrieved. After eliminating 137 duplicates, the remaining records underwent a two-step screening process. In the first step, 141 studies were excluded after reviewing their titles. Next, a thorough examination of abstracts led to the exclusion of 14 more studies. This left five studies for full-text assessment, out of which five met the eligibility criteria and were included in the final meta-analysis. The detailed process is illustrated in Figure 1.

### 3.2. Characteristics of Study and Participant

The main characteristics of the RCTs included in the final analysis are presented in Table 1. A total of 439 adult participants were enrolled, with 220 allocated to the remimazolam group and 219 to the dexmedetomidine group. Four studies [10,11,12,13] investigated patients undergoing orthopedic surgeries (e.g., knee or hip arthroplasty, osteotomy, and meniscectomy), while the remaining study [14] included individuals undergoing any type of surgery performed under regional anesthesia. Sedation depth was measured using the Bispectral Index (BIS) in two studies [10,11], while the remaining three studies utilized the Modified Observer’s Assessment of Alertness/Sedation (MOAA/S) scale [12,13,14]. Different dosages of remimazolam and dexmedetomidine were administered across the studies. Various definitions for respiratory depression, bradycardia, hypotension, and hypertension were used in each study, as shown in Supplementary Table S2.

### 3.3. Incidence of Respiratory Depression

**Figure 2:**
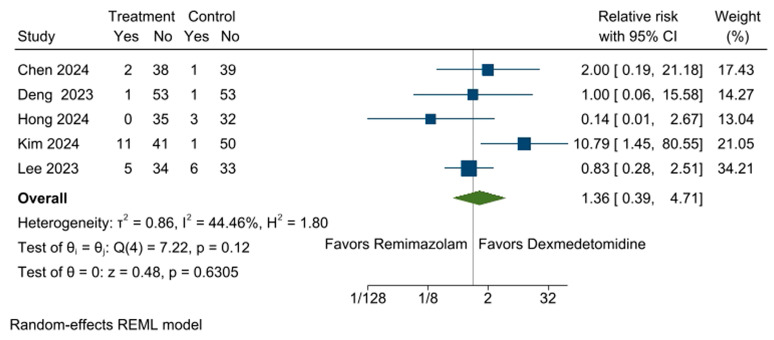
Forest plot for the incidence of respiratory depression during sedation. No significant difference was observed in the incidence of respiratory depression between the remimazolam and dexmedetomidine groups. CI: confidence interval [10,11,12,13,14].

The incidence of respiratory depression during sedation was reported in five RCTs [10,11,12,13,14]. The incidence of respiratory depression was 8.6% (19 out of 220 participants) in the remimazolam group and 5.5% (12 out of 219 participants) in the dexmedetomidine group. The overall pooled effect size revealed that remimazolam administration did not increase the risk of intraoperative respiratory depression during sedation (RR: 1.36, 95% CI [0.39, 4.71], *p* = 0.6305, I^2^ = 44%; Figure 2). The sensitivity analysis revealed that excluding any single study did not significantly alter the pooled RR, confirming the stability and reliability of our findings (Supplementary Figure S1).

### 3.4. Incidence of Bradycardia

**Figure 3:**
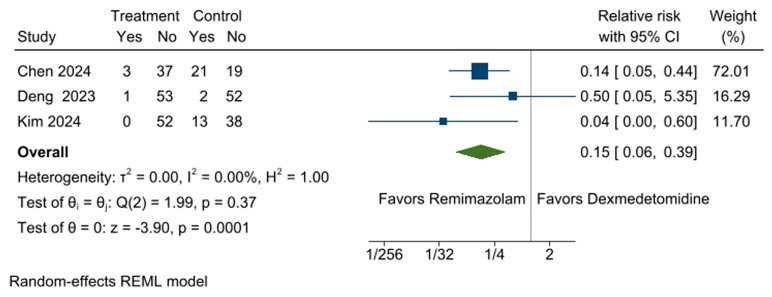
Forest plot for the incidence of bradycardia during surgery. A significantly lower incidence of bradycardia was observed in the remimazolam group compared to the dexmedetomidine group. CI: confidence interval [10,11,13].

Three RCTs [10,11,13] were included in the meta-analysis for the incidence of bradycardia between the remimazolam and dexmedetomidine groups. Participants in the remimazolam group (2.7%, 4 out of 146 participants) had a lower incidence of bradycardia compared to the dexmedetomidine group (24.8%, 36 out of 145 participants) (RR: 0.15, 95% CI [0.06, 0.39], *p* = 0.0001, I^2^ = 0%; Figure 3). The sensitivity analysis using the leave-one-out approach showed that the significance of the effect size for bradycardia incidence changed when a specific study [10] was excluded, suggesting that the result was influenced by the inclusion of this study (Supplementary Figure S2).

### 3.5. Incidence of Hypotension

**Figure 4:**
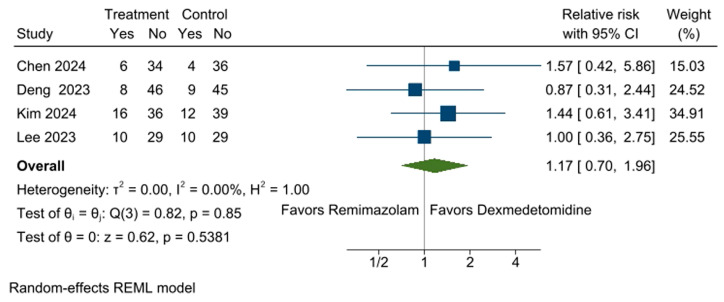
Forest plot for the incidence of hypotension. Comparable incidences of intraoperative hypotension were observed between the remimazolam and dexmedetomidine groups. CI: confidence interval [10,11,13,14].

Four studies [10,11,13,14] reported the incidence of hypotension, which occurred in 21.6% (40 out of 185 participants) of the remimazolam group and 19.0% (35 out of 184 participants) of the dexmedetomidine group. The pooled analysis indicated that remimazolam did not significantly increase the risk of intraoperative hypotension in patients undergoing surgery with dexmedetomidine sedation (RR: 1.17, 95% CI [0.70, 1.96], *p* = 0.5381, I^2^ = 0%; Figure 4). In the leave-one-out sensitivity analysis, no study skewed the effect size, supporting the robustness of the present results (Supplementary Figure S3).

### 3.6. Incidence of Hypertension

**Figure 5:**
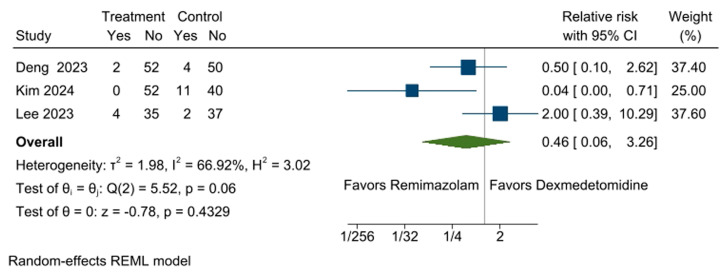
Forest plot for the incidence of hypertension. Participants in the remimazolam group showed a similar incidence of hypertension compared to those in the dexmedetomidine group. CI: confidence interval [11,13,14].

Incidence of hypertension was assessed in three RCTs [11,13,14]. No significant difference was observed between the remimazolam (4.1%, 6 out of 145 participants) and dexmedetomidine groups (11.8%, 17 out of 144 participants) (RR: 0.46, 95% CI [0.06, 3.26], *p* = 0.4329, I^2^ = 67%; Figure 5). Leave-one-out sensitivity analysis did not reveal changes in the pooled effect size of the incidence of hypertension (Supplementary Figure S4).

### 3.7. Respiratory Rates During Sedation

Three RCTs [10,12,14] evaluated respiratory rate, showing no notable difference between the remimazolam and the dexmedetomidine groups (MD: –0.36, 95% CI [–0.95, 0.24], *p* = 0.2390, I^2^ = 0%; Table 2). Sensitivity analysis using the leave-one-out method indicated no variation in the pooled effect size for respiratory rate (Supplementary Figure S5).

### 3.8. Heart Rates During Sedation

Heart rates were assessed in three trials [10,12,14], and the pooled analysis demonstrated that heart rates were significantly higher in the remimazolam group compared to the dexmedetomidine group (MD: 8.17, 95% CI [6.32, 10.03], *p* = 0.0000, I^2^ = 0%; Table 2). Sensitivity analysis using the leave-one-out method showed no changes in the effect size when individual studies were excluded (Supplementary Figure S6).

### 3.9. Mean Arterial Pressure During Sedation

In three studies [10,12,14] comparing remimazolam and dexmedetomidine, the mean arterial pressure was investigated. Regardless of whether remimazolam or dexmedetomidine was administered, participants had similar mean arterial pressure (MD: 9.01, 95% CI [–9.97, 27.99], *p* = 0.3522, I^2^ = 99%; Table 2). In the sensitivity analysis, no small study effects were detected, and the effect size remained stable (Supplementary Figure S7).

### 3.10. Time to Target Sedation Depth

Three RCTs [10,12,13] were included in the data synthesis for the time to reach target sedation depth between the remimazolam and dexmedetomidine groups. Participants in the remimazolam group achieved the desired level of sedation more quickly compared to those in the dexmedetomidine group (MD: −6.04, 95% CI [−6.99, −5.09], *p* = 0.0000, I^2^ = 68%; Table 2). Sensitivity analysis using the leave-one-out approach showed that the significance of the effect size for time to target sedation depth remained consistent regardless of which study was excluded (Supplementary Figure S8).

### 3.11. Emergence Time from Sedation

Three RCTs [10,12,13] reported the emergence time from sedation, showing no significant difference between the remimazolam and dexmedetomidine groups (MD: −11.84, 95% CI [−25.87, 2.19], *p* = 0.0981, I^2^ = 99%; Table 2). In the sensitivity analysis using the leave-one-out method, the significance of the effect size for the emergence time from sedation was altered when one study [13] were omitted, indicating that the result was sensitive to the inclusion of this particular study (Supplementary Figure S9).

### 3.12. Incidence of PONV

Four studies [11,12,13,14] evaluated the occurrence of PONV. The remimazolam group showed a pooled incidence of PONV at 11.1% (20 out of 180 participants), while the dexmedetomidine group had a rate of 10.1% (18 out of 179 participants), indicating no significant difference between the two groups (RR: 1.27, 95% CI [0.68, 2.37], *p* = 0.4535, I^2^ = 0%; Table 2). The pooled effect size remained consistent in the sensitivity analysis, supporting the robustness of our results (Supplementary Figure S10).

### 3.13. Risk of Bias

Supplementary Figures S11 and S12 provide an overview of the overall risk of bias. Most studies included in this meta-analysis were deemed to have a “low” risk of bias [11,12,13,14]. However, one study [10] was assessed as having “some concerns” due to inadequate reporting on group allocation concealment before enrollment and intervention and insufficient details regarding blinding during postoperative outcome evaluation.

### 3.14. Certainty of Evidence

The assessment of evidence certainty is presented in Supplementary Table S3. High certainty of evidence was attributed to the outcomes for the incidence of hypertension and PONV. Six outcomes—respiratory depression, bradycardia, hypotension, respiratory rates, heart rates, and time to achieve target sedation depth—were determined to have moderate certainty, primarily influenced by concerns related to the risk of bias. Conversely, the remaining outcomes, including mean arterial pressure and emergence time from sedation, were rated as having low certainty of evidence due to significant heterogeneity and identified concerns regarding potential bias.

## 4. Discussion

This meta-analysis provides an in-depth comparison of remimazolam and dexmedetomidine as sedative agents for patients undergoing regional anesthesia, focusing on their effects on respiratory function, hemodynamic stability, sedation efficacy, and adverse events. The findings offer valuable evidence for clinicians to guide the selection of sedative agents, addressing both efficacy and safety profiles.

The results indicate that remimazolam is as safe as dexmedetomidine regarding respiratory depression during sedation. The observed incidence of respiratory depression was low in both groups (8.6% and 5.5% in the remimazolam and the dexmedetomidine group, respectively), and the pooled effect size showed no significant difference. This finding supports the use of remimazolam as a viable sedative agent, particularly in patients at higher risk for respiratory complications. As a benzodiazepine derivative, remimazolam is rapidly metabolized by nonspecific tissue esterase [4], contributing to its favorable respiratory safety profile. These results reinforce previous studies suggesting that remimazolam is less likely to compromise respiratory function compared to other sedatives, such as propofol [15].

The hemodynamic effects of the two agents were a central focus of this analysis. A key finding was the significantly lower incidence of bradycardia in the remimazolam group (2.7%) compared to the dexmedetomidine group (24.8%). Dexmedetomidine, an α_2_-adrenergic receptor agonist, is known for its potential to induce bradycardia due to its mechanism of action [5]. In contrast, remimazolam exhibited superior hemodynamic stability, making it a safer option for patients with pre-existing cardiovascular conditions. While the rates of hypotension and hypertension were comparable between the two groups, this demonstrates the importance of monitoring blood pressure closely during sedation, regardless of the agent used.

Remimazolam demonstrated a significant advantage in achieving the target sedation depth more rapidly than dexmedetomidine (about 6 min). This faster onset is particularly beneficial in time-sensitive surgical settings where prompt sedation initiation is critical. Despite the differences in sedation onset, the emergence times from sedation were not statistically different between the two groups. However, the mean difference in recovery time was approximately 12 min, indicating a potential clinical advantage for one agent in facilitating a slightly faster recovery once sedation is discontinued. These findings highlight the potential of remimazolam as a practical sedative agent, offering a balance between rapid onset and a manageable recovery profile.

The analysis showed no significant difference in the incidence of PONV between remimazolam and dexmedetomidine. This result is clinically relevant, as PONV can negatively impact patient satisfaction and recovery [16]. Both agents exhibited similar tolerability regarding this adverse event, making them comparable in terms of postoperative patient comfort.

The results of this analysis demonstrate the clinical utility of both remimazolam and dexmedetomidine as sedative agents, each offering distinct advantages. The rapid onset of remimazolam and its lower risk of bradycardia make it an attractive choice in settings where hemodynamic stability and prompt sedation are priorities. On the other hand, dexmedetomidine remains valuable for its sedative and mild analgesic properties [5]. The personalized selection of these agents should consider individual patient characteristics, surgical context, and potential risks. Future research should address the identified limitations by including larger, multicenter randomized controlled trials to confirm these findings. Additionally, studies directly comparing remimazolam and dexmedetomidine in specific surgical populations, such as elderly patients or those with pre-existing cardiovascular or respiratory conditions, would provide more targeted evidence to guide clinical practice.

This meta-analysis employed rigorous methods, including a comprehensive search strategy, predefined inclusion criteria, and robust statistical techniques. Sensitivity analyses further validated the stability of the results, adding confidence to the findings. However, several limitations must be acknowledged. First, the number of included studies was limited for certain outcomes, such as hypertension and emergence time, potentially reducing the statistical power of the analysis. Additionally, the small number of eligible studies and total sample size may limit the generalizability of this meta-analysis. Although some results suggest potential benefits of remimazolam, this should not be interpreted as conclusive evidence of superiority. The findings must be considered exploratory and hypothesis-generating, and further high-quality, large-scale RCTs are needed to validate these observations. Second, a significant limitation of this meta-analysis is the heterogeneity in the definitions of several key outcome variables across the included studies. For example, the criteria for “respiratory depression”, “hypotension”, and “hypertension” varied among studies, with different thresholds for oxygen saturation or blood pressure used. These inconsistencies may have contributed to inter-study variability and introduced potential bias in the pooled analyses. Future studies would benefit from standardized outcome definitions to improve comparability and reliability. Third, this meta-analysis predominantly includes studies conducted in Korea and China, which may limit the generalizability of the findings to other regions. Differences in healthcare systems, cultural practices, surgical protocols, anesthesia techniques, and patient demographics in these countries may influence the results. Consequently, caution should be exercised when applying these findings to populations in other geographic areas with distinct clinical settings or practices. Fourth, the studies included in this analysis evaluated sedation depth using two different methods: the BIS and the MOAA/S scale. BIS, which quantifies sedation depth based on electroencephalographic waveforms, allows for objective titration of sedative agents. However, given the reported linear correlation between BIS and the MOAA/S scale [17], it is unlikely that the use of MOAA/S introduced significant variability in the assessment or management of sedation depth in this meta-analysis. Fifth, one included study [11] had a mean patient age above 70 years. While the European Medicines Agency recommends caution with dexmedetomidine use in ICU patients over 65 due to cardiovascular risks such as bradycardia and hypotension, our meta-analysis focused on surgical patients under regional anesthesia, not ICU patients. Interestingly, some studies have reported increased mortality in ICU patients under 65 years old, potentially due to confounding factors such as illness severity or selection bias [18,19]. Nevertheless, caution is still necessary when interpreting results involving older patients, as age-related differences in the safety profile of dexmedetomidine may exist. The direction and magnitude of any age-related bias in our findings remain unclear. Sixth, of note, one study defined respiratory depression as a decrease in SpO_2_ below 93% [13], whereas others used a more conservative threshold of 90%. This discrepancy in definition may have contributed to the finding that only one study reported a significant difference in respiratory depression between the treatment groups. Such variability underscores the need for standardized definitions in future research. Lastly, most of the studies included involved patients undergoing orthopedic surgeries, which may limit generalizability to other surgical contexts. This potential selection bias should be considered when applying the findings to broader populations.

Several questions remain unresolved, such as the optimal dosing strategies for remimazolam in various surgical contexts, its effects in high-risk or elderly populations, and its interaction with other perioperative medications. Future studies should also aim to standardize outcome definitions, particularly for respiratory depression and hemodynamic parameters, to facilitate more robust comparisons across studies.

## 5. Conclusions

In conclusion, this meta-analysis demonstrates that remimazolam is a safe and effective alternative to dexmedetomidine for sedation during regional anesthesia. Remimazolam offers significant advantages, including a lower risk of bradycardia, faster onset of sedation, and comparable efficacy in maintaining sedation depth and recovery profiles. Both agents exhibited similar safety profiles in terms of respiratory outcomes, hemodynamic stability, and the incidence of PONV, making them viable choices depending on the clinical context. Further research, particularly large-scale trials focused on specific patient populations, is needed to confirm these results and explore long-term outcomes, ensuring optimal patient care and recovery.

## Figures and Tables

**Table 1:** Characteristics of the studies included.

Author	Year	Procedure	Sample Size		Age		Assessment Tools	Induction		Maintenance	
			N1	N2	A1	A2		Remimazolam	Dexmedetomidine	Remimazolam	Dexmedetomidine
Chen [10]	2024	Orthopedic surgery	40	40	58.8	61.7	Ramsay score of 2–5 and BIS 60–80	0.03 mg/kg/min	0.3 µg/kg	0.2–0.5 mg/kg/h	0.2–1.0 µg/kg/h
Deng [11]	2023	Total joint arthroplasty	54	54	70.8	71.8	BIS 70–80	0.025–0.1 mg/kg	0.2–0.7 µg/kg/h	0.1–1.0 mg/kg/h	0.2–0.7 µg/kg/h
Hong [12]	2024	Orthopedic surgery	35	35	39.0	38.0	MOAA/S 3	6 mg/kg/h	6 µg/kg/h	1 mg/kg/h	1 µg/kg/h
Kim [13]	2024	Orthopedic surgery	52	51	52.0	54.0	MOAA/S 3 or 4	0.075 mg/kg	1 µg/kg	0.5–1 mg/kg/h	0.2–0.7 µg/kg/h
Lee [14]	2023	Surgery under regional anesthesia	39	39	61.1	57.3	MOAA/S 3	2.5 mg	1 µg/kg	0.1–1.0 mg/kg/h	0.2–0.7 µg/kg/h

A1: mean age in remimazolam group; A2: mean age in the dexmedetomidine group; N1: number of participants in the remimazolam group; N2: number of participants in the dexmedetomidine group. BIS: Bispectral Index; Modified Observer’s Assessment of Alertness/Sedation: MOAA/S.

**Table 2:** Results of pooled effect size.

	No. of Studies	No. of Participants	MD (95% CI)	*p*-Value	I^2^
Respiratory rates	3	228	–0.36 [–0.95, 0.24]	0.2390	0%
Heart rates	3	228	8.17 [6.32, 10.03]	0.0000	0%
Mean arterial pressure	3	228	9.01 [–9.97, 27.99]	0.3522	99%
Time to target sedation depth	3	253	–6.04 [–6.99, –5.09]	0.0000	68%
Emergence time from sedation	3	253	–11.84 [–25.87, 2.19]	0.0981	99%
PONV	4	359	1.27 [0.68, 2.37]	0.4535	0%

CI: confidence interval; MD: mean difference; PONV: postoperative nausea and vomiting.

## Data Availability

The original contributions presented in this study are included in the article/Supplementary Materials. Further inquiries can be directed to the corresponding author.

## Supplementary Materials

The following supporting information can be downloaded at: https://www.mdpi.com/article/10.3390/medicina61040726/s1.
