# Supplementary material for: A Comparison of the Safety and Efficacy of Remimazolam and Dexmedetomidine for Sedation in Surgical Patients Under Regional Anesthesia: A Meta-Analysis of Randomized Controlled Trials
Source: Medicina (Kaunas). 2025 Apr 14;61(4):726. doi: 10.3390/medicina61040726 (PMC12028552; doi:10.3390/medicina61040726)
Supplement: Supplementary file 1 [file medicina-61-00726-s001.zip › medicina-3541249-supplementary.pdf]

Supplementary Files

Table S1. Search strategy for each database

Database	Order	Keywords	Results
PubMed	#1	"Remimazolam"[Title/Abstract] OR "Byfavo"[Title/Abstract] OR "CNS-7056"[Title/Abstract]	750
	#2	"Dexmedetomidine"[Title/Abstract] OR "Precedex"[Title/Abstract] OR "Dexdor"[Title/Abstract] OR "Dexdomitor"[Title/Abstract] OR "Dexmed"[Title/Abstract] OR "Dexem"[Title/Abstract] OR "Dexit"[Title/Abstract]	9,861
	#3	"sedation"[Title/Abstract]	50,616
	#4	#1 AND #2	54
	#5	#3 AND #4	**39**
EMBASE	#1	remimazolam:ti,ab,kw OR byfavo:ti,ab,kw OR 'cns 7056':ti,ab,kw	779
	#2	dexmedetomidine:ti,ab,kw OR precedex:ti,ab,kw OR dexdor:ti,ab,kw OR dexmedesed:ti,ab,kw OR dexdomitor:ti,ab,kw OR dexmed:ti,ab,kw OR dexanest:ti,ab,kw OR dextomid:ti,ab,kw OR dexmeto:ti,ab,kw OR dexem:ti,ab,kw OR dexit:ti,ab,kw	14,366
	#3	sedation:ti,ab,kw	80,857
	#4	#1 AND #2	52
	#5	#3 AND #4	**39**
CENTRAL	#1	(Remimazolam OR Byfavo OR CNS-7056):ti,ab,kw	1,063
	#2	(Dexmedetomidine OR Precedex OR Dexdor OR Dexmedesed OR Dexdomitor OR Dexmed OR Dexanest OR Dextomid OR Dexmeto OR Dexem OR Dexit):ti,ab,kw	10,411
	#3	(Sedation):ti,ab,kw	33,237
	#4	#1 AND #2	81
	#5	#3 AND #4	**68**
SCOPUS	#1	TITLE-ABS-KEY (remimazolam OR byfavo OR cns-7056)	911
	#2	TITLE-ABS-KEY (dexmedetomidine OR precedex OR dexdor OR dexmedesed OR dexdomitor OR dexmed OR dexanest OR dextomid OR dexmeto OR dexem OR dexit)	19,644
	#3	TITLE-ABS-KEY (sedation)	102,970
	#4	#1 AND #2	119
	#5	#3 AND #4	**86**
Web of Science	#1	TS=(Remimazolam OR Byfavo OR CNS-705)	756
	#2	(TS=(Remimazolam OR Byfavo OR CNS-705)) AND TS=(Dexmedetomidine OR Precedex OR Dexdor OR Dexmedesed OR Dexdomitor OR Dexmed OR Dexanest OR Dextomid OR Dexmeto OR Dexem OR Dexit)	12,434
	#3	TS=(Sedation)	52,201
	#4	#1 AND #2	79
	#5	#3 AND #4	**65**

Table S2. Definition of hemodynamic variables

Study	Definition
**Respiratory depression**	
Chen 2024	SpO_2 < 90%
Deng 2023	SpO_2 < 90% or an absolute decrease of > 5% from baseline
Hong 2024	SpO_2 < 90%
Kim 2024	SpO_2 <93% or a respiratory rate of <8 breaths/min
Lee 2023	Decrease in SpO_2 due to the cessation of spontaneous breathing
**Bradycardia**	
Chen 2024	Heart rate less than 50 beats/minute or less than 20% of the baseline value
Deng 2023	Heart rate < 50 beats/minute or a decrease of > 20% from baseline
Hong 2024	Heart rate < 40 beats/minute
Kim 2024	Heart rate < 45 beats/minute
Lee 2023	No definition
**Hypotension**	
Chen 2024	A decrease in systolic blood pressure of over 20% less than the baseline value
Deng 2023	Systolic blood pressure < 90 mmHg or a decrease of > 20% from baseline
Hong 2024	Mean arterial pressure < 60 mmHg
Kim 2024	Mean arterial pressure < 65 mmHg
Lee 2023	A decrease of > 20% from baseline
**Hypertension**	
Chen 2024	No definition
Deng 2023	Systolic blood pressure > 160 mmHg or an increase of > 20% from baseline
Hong 2024	No definition
Kim 2024	Systolic blood pressure of >120% of the baseline
Lee 2023	Systolic blood pressure of >120% of the baseline

Table S3. Certainty for each outcome

Certainty assessment							№ of patients		Effect		Certainty	Importance
№ of studies	Study design	Risk of bias	Inconsistency	Indirectness	Imprecision	Other considerations	RMZ	Dexmedetomidine	Relative (95% CI)	Absolute (95% CI)		
**Respiratory depression**												
5	randomised trials	serious[a]	not serious	not serious	not serious	none	19/220 (8.6%)	12/219 (5.5%)	**RR 1.36** (0.39 to 4.71)	**20 more per 1,000** (from 33 fewer to 203 more)	⨁⨁⨁◯ Moderate[a]	
**Bradycardia**												
3	randomised trials	serious[a]	not serious	not serious	not serious	none	4/146 (2.7%)	36/145 (24.8%)	**RR 0.15** (0.06 to 0.39)	**211 fewer per 1,000** (from 233 fewer to 151 fewer)	⨁⨁⨁◯ Moderate[a]	
**Hypotension**												

Certainty assessment							№ of patients		Effect		Certainty	Importance
№ of studies	Study design	Risk of bias	Inconsistency	Indirectness	Imprecision	Other considerations	RMZ	Dexmedetomidine	Relative (95% CI)	Absolute (95% CI)		
4	randomised trials	serious[a]	not serious	not serious	not serious	none	40/185 (21.6%)	35/184 (19.0%)	**RR 1.17** (0.70 to 1.96)	**32 more per 1,000** (from 57 fewer to 183 more)	⊕⊕⊕◯ Moderate[a]	
**Hypertension**												
3	randomised trials	not serious	not serious	not serious	not serious	none	6/145 (4.1%)	17/144 (11.8%)	**RR 0.46** (0.06 to 3.26)	**64 fewer per 1,000** (from 111 fewer to 267 more)	⊕⊕⊕⊕ High	
**Respiratory rate**												
3	randomised trials	serious[a]	not serious	not serious	not serious	none			-	MD **0.36 fewer** (0.95 fewer to 0.24 more)	⊕⊕⊕◯ Moderate[a]	
**Heart rate**												

Certainty assessment							№ of patients		Effect		Certainty	Importance
№ of studies	Study design	Risk of bias	Inconsistency	Indirectness	Imprecision	Other considerations	RMZ	Dexmedetomidine	Relative (95% CI)	Absolute (95% CI)		
3	randomised trials	serious[a]	not serious	not serious	not serious	none			-	MD **8.17 more** (6.23 more to 10.03 more)	⊕⊕⊕◯ Moderate[a]	
**Mean arterial pressure**												
3	randomised trials	serious[a]	serious[b]	not serious	not serious	none			-	MD **9.01 higher** (9.97 lower to 27.99 higher)	⊕⊕◯◯ Low[a,b]	
**Time to target sedation depth**												
3	randomised trials	serious[a]	not serious	not serious	not serious	none			-	MD **6.04 lower** (6.99 lower to 5.09 lower)	⊕⊕⊕◯ Moderate[a]	
**Emergence time from sedation**												

Certainty assessment							№ of patients		Effect		Certainty	Importance
№ of studies	Study design	Risk of bias	Inconsistency	Indirectness	Imprecision	Other considerations	RMZ	Dexmedetomidine	Relative (95% CI)	Absolute (95% CI)		
3	randomised trials	serious[a]	serious[b]	not serious	not serious	none			-	MD **11.84 lower** (25.87 lower to 2.19 higher)	⨁⨁◯◯ Low[a,b]	
**Postoperative nausea and vomiting**												
4	randomised trials	not serious	not serious	not serious	not serious	none	20/180 (11.1%)	18/179 (10.1%)	**RR 1.27** (0.68 to 2.37)	**27 more per 1,000** (from 32 fewer to 138 more)	⨁⨁⨁⨁ High	

CI: confidence interval; **MD:** mean difference; **OR:** odds ratio

Explanations

a. Downgrade for some concerns in risk of bias

b. Downgrade for inconsistency (High heterogeneity)

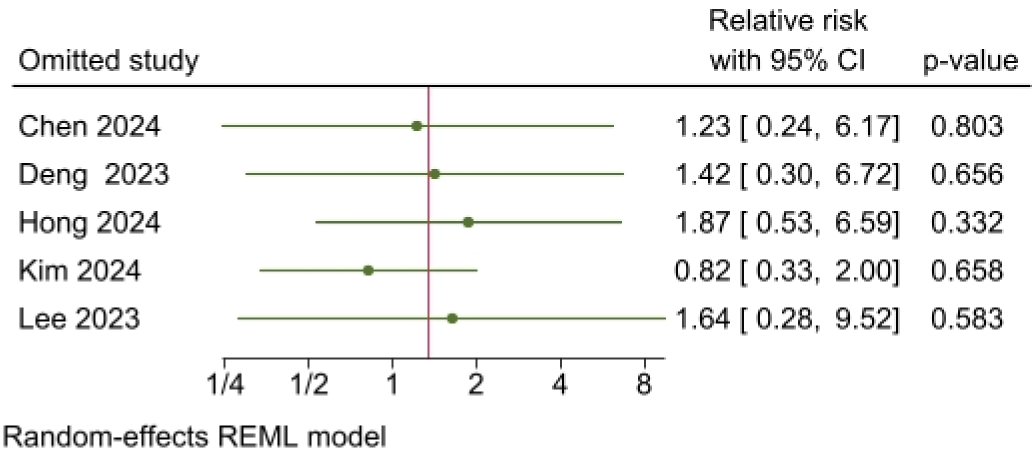

Figure S1. Forest plot for sensitivity analysis of the incidence of respiratory depression in the comparison between remimazolam and dexmedetomidine groups. Sensitivity analysis identified no alteration of effect size. CI: confidence interval.

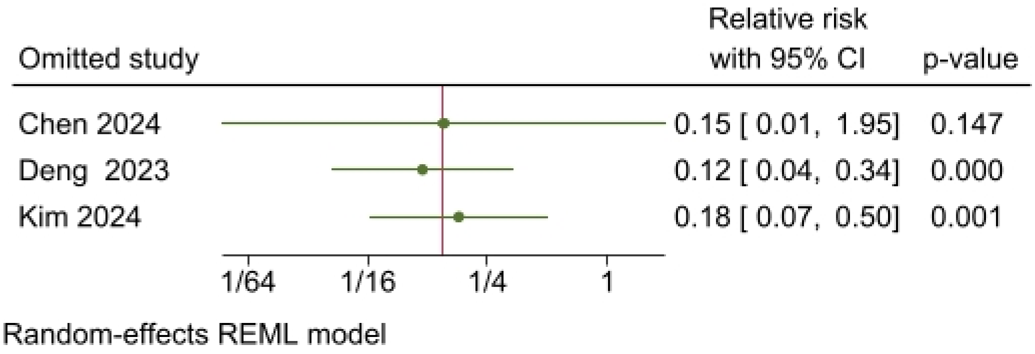

Figure S2. Forest plot for the sensitivity analysis of the incidence of bradycardia comparing the remimazolam and dexmedetomidine groups. The sensitivity analysis revealed a change in the effect size upon omitting one study (Chen, 2024). CI: confidence interval.

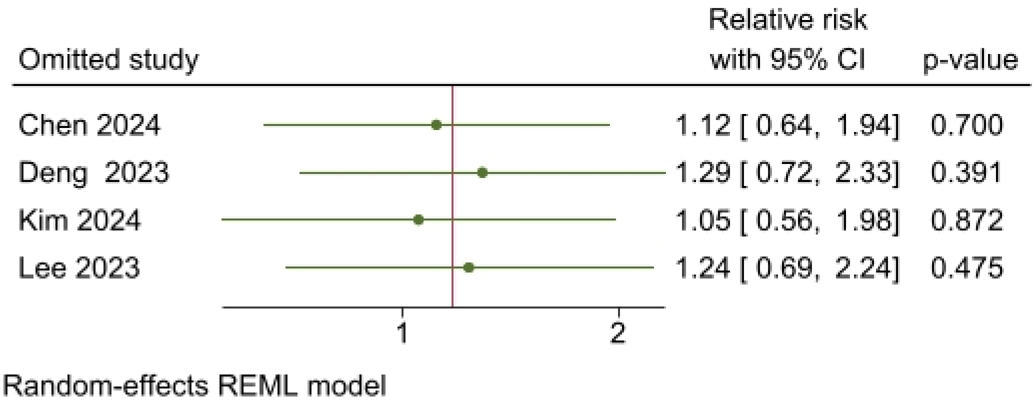

Figure S3. Forest plot for sensitivity analysis of incidence of hypotension. The sensitivity analysis did not show any change in the significance of the pooled effect size. CI: confidence interval.

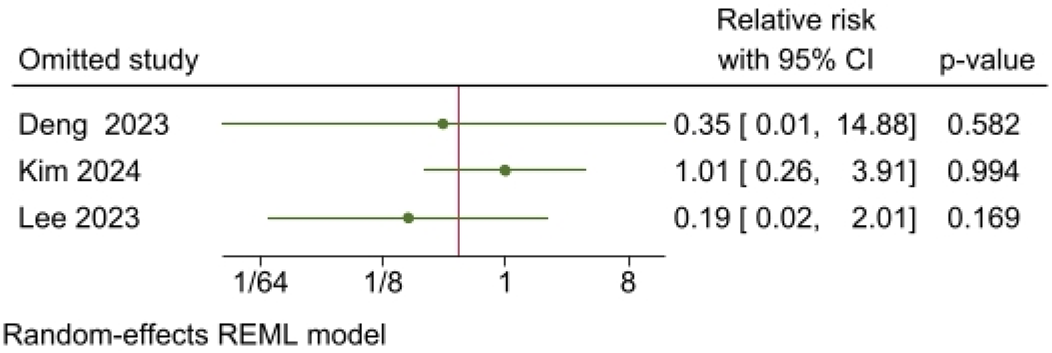

Figure S4. Forest plot for sensitivity analysis of the incidence of hypertension. No meaningful changes were observed in the effect size. CI: confidence interval.

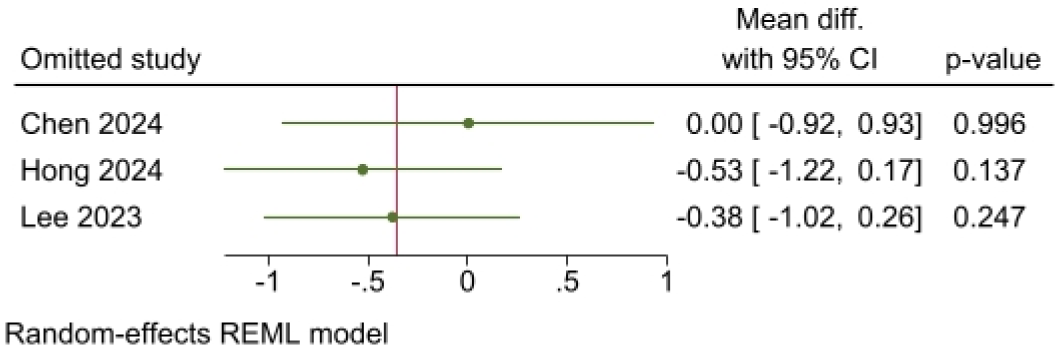

Figure S5. Forest plot for sensitivity analysis of respiratory rates. No meaningful changes were observed in the effect size. CI: confidence interval.

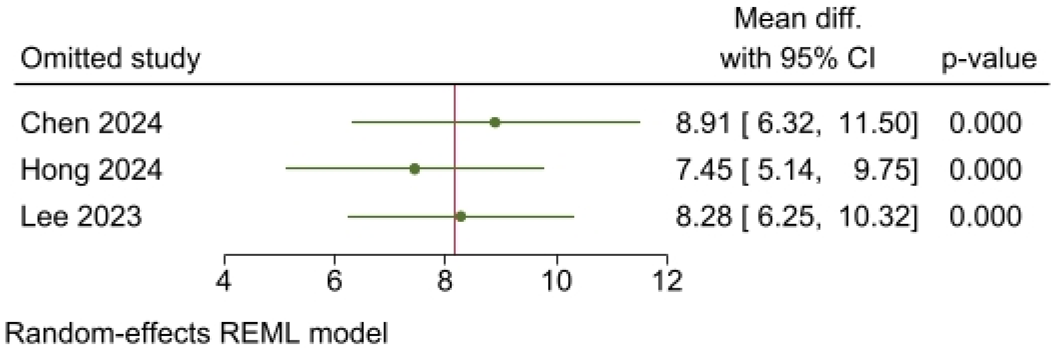

Figure S6. Forest plot for sensitivity analysis of heart rates. Sensitivity analysis showed no effect size changes by omitting studies. CI: confidence interval.

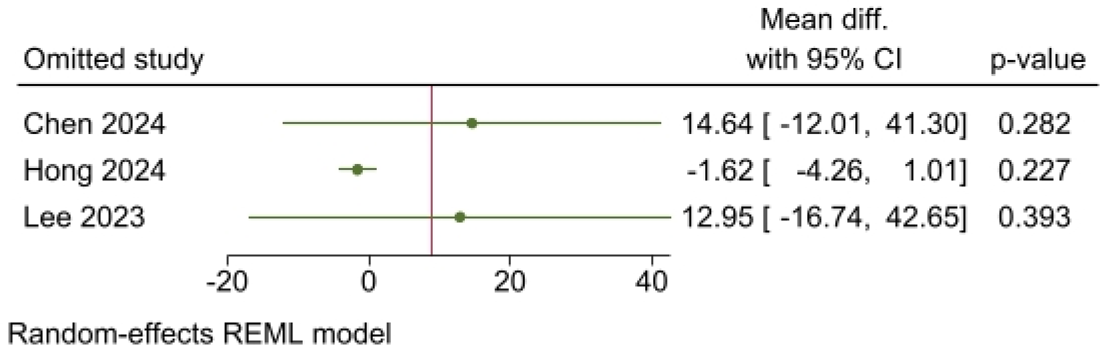

Figure. S7. Forest plot for sensitivity analysis of mean arterial pressure. Sensitivity analysis showed no effect size changes by omitting studies. CI: confidence interval.

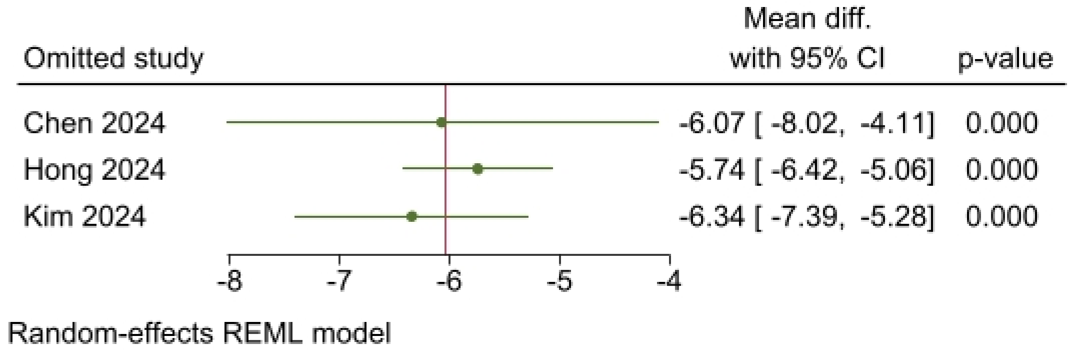

Figure S8. Forest plot for sensitivity analysis of time to target sedation depth. Sensitivity analysis showed no effect size changes by omitting studies. CI: confidence interval.

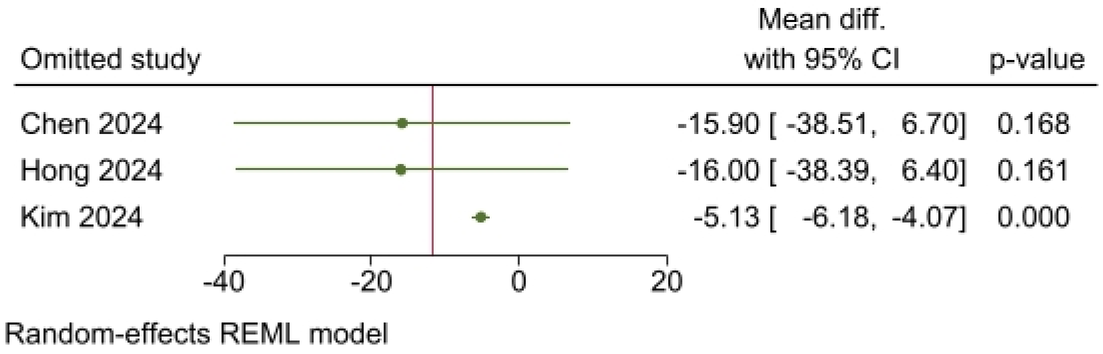

Figure S9. Forest plot for sensitivity analysis of emergence time from sedation. Sensitivity analysis revealed a change in the effect size upon omitting one study (Kim 2024). CI: confidence interval.

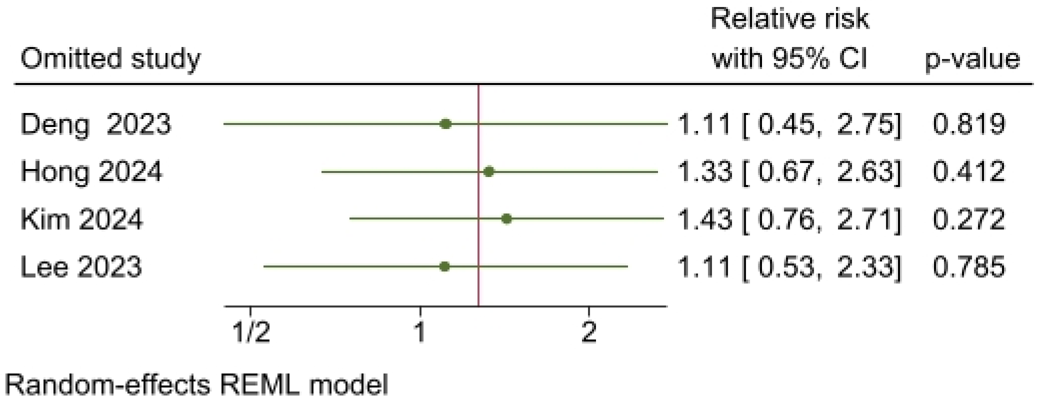

Figure S10. Forest plot for sensitivity analysis of postoperative nausea and vomiting. Sensitivity analysis showed no effect size changes by omitting studies. CI: confidence interval.

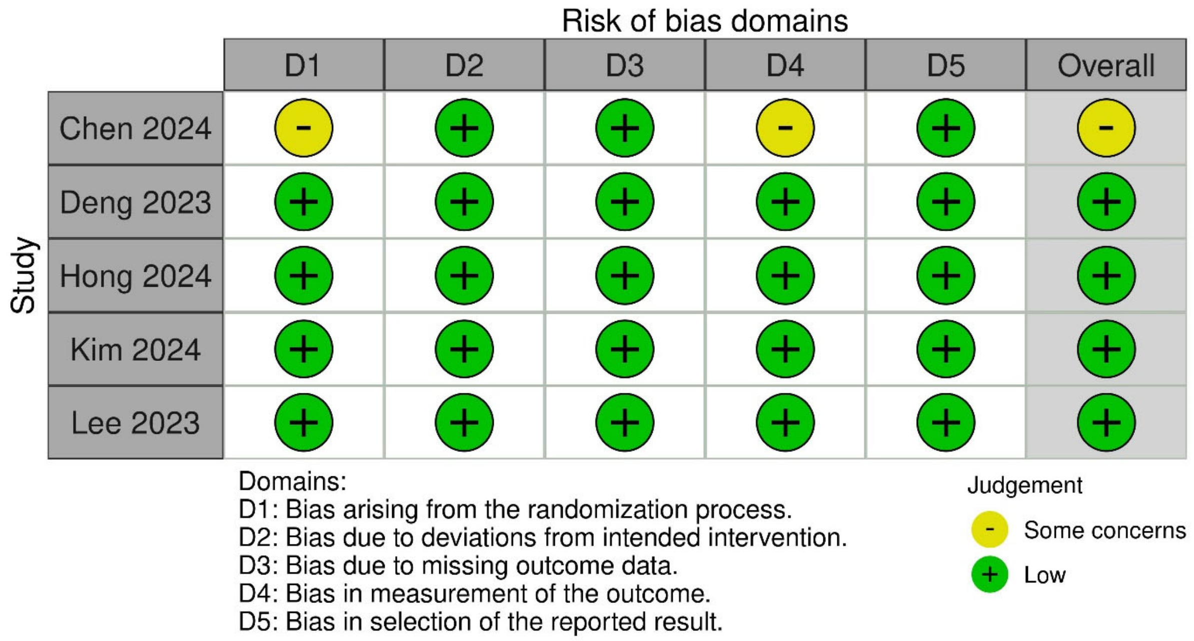

Figure S11. Risk of bias summary. Green circle, low risk; yellow circle, some concerns; red circle, high risk

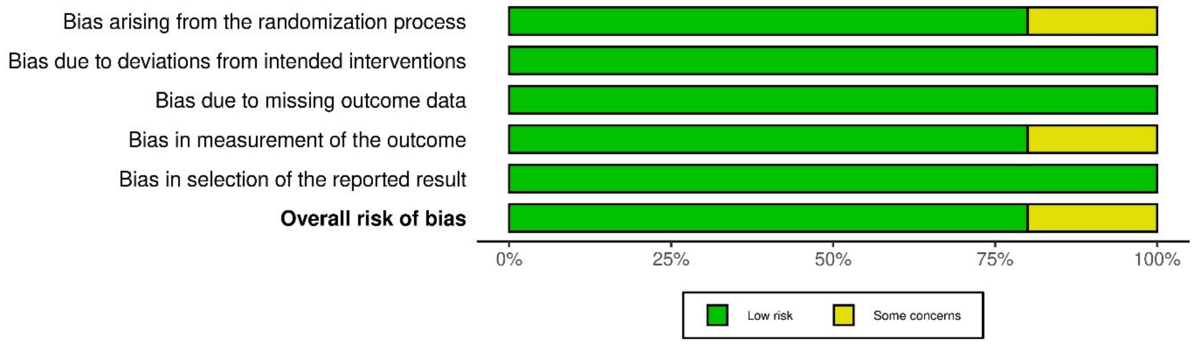

Figure. S12. Overall risk of bias as a summary plot.
